# Advancing diagnostics in suspected periprosthetic joint infections using synthetic synovial fluid and microcalorimetry

**DOI:** 10.5194/jbji-11-149-2026

**Published:** 2026-03-11

**Authors:** Amber De Bleeckere, Jeroen Neyt, Jasper Van Heuverswyn, Stien Vandendriessche, Hannelore Hamerlinck, Annelynn Wallaert, Christophe Pattyn, Bruno Verhasselt, Jerina Boelens, Tom Coenye

**Affiliations:** 1 Laboratory of Pharmaceutical Microbiology, Ghent University, Ghent, Belgium; 2 Department of Orthopedic Surgery and Traumatology, Ghent University Hospital, Ghent, Belgium; 3 Department of Medical Microbiology, Ghent University Hospital, Ghent, Belgium; 4 Department of Diagnostic Sciences, Ghent University, Ghent, Belgium

## Abstract

Rapid and accurate pathogen detection is essential for effective management of periprosthetic joint infections (PJIs), yet conventional culturing (CC) often yields false-negative results and requires prolonged incubation times. In the present study we compared the performance of CC to that of two alternative approaches, i.e., culturing in synthetic synovial fluid (SSF2) and isothermal microcalorimetry (IMC).

A total of 79 synovial fluid (SF) samples from patients with suspected PJI were included; for these samples, CC data were available. Samples were incubated in SSF2 (aerobically and anaerobically, for 10 d), and isolates were identified by matrix-assisted laser desorption/ionization mass spectrometry (MALDI-TOF MS). With IMC we determined the time to detect microbial activity in the samples (in two different media; brain heart infusion (BHI) broth and fluid thioglycolate medium (FTM)).

Culturing in SSF2 yielded the highest positivity rate (53.2 %), followed by IMC and CC (34.2 % and 32.9 %, respectively). More than one-third of all positive samples were detected only after culturing in SSF2 (39.3 %), and this approach also revealed the greatest microbial diversity. IMC enabled rapid detection of microbial activity in a sample, with median detection times of 15.9 h in BHI and 15.6 h in FTM.

Our results demonstrate that culturing of SF samples in SSF2 increased the diagnostic yield and that IMC reduced the time to identify clinical samples that contain viable microorganisms. This highlights the potential of these approaches; however further optimization is warranted to integrate them in diagnostic PJI workflows.

## 1 Introduction

Effective management of PJI requires accurate and fast detection of causative pathogens to guide targeted antimicrobial therapy (Patel, 2023). The diagnosis of a PJI needs to be confirmed using a number of criteria, including microbiological tests to detect the presence of a pathogen (McNally et al., 2021). Currently, this relies mostly on conventional culturing (CC) (Parvizi et al., 2018), which is time-consuming (requiring up to 14 d of incubation for fastidious microorganisms) and may yield false-negative results (approximately 7 %–15 % of cases) (Berbari et al., 2007; Palan et al., 2019). False-negative results are often attributed to prior administration of antibiotics, while a culture-negative result in the absence of signs of infection may suggest “aseptic loosening”. However, a considerable fraction of cases of aseptic loosening may actually represent low-grade infection with fastidious pathogens undetected by CC (Nelson et al., 2005).

To reduce the number of false-negative results, mimicking the infectious microenvironment in in vitro testing has been advocated (Bjarnsholt et al., 2013, 2022), as it has been demonstrated that bacteria exhibit a distinct gene expression profile in vitro versus in vivo and thus behave differently in distinct environments (Cornforth et al., 2018; Xu et al., 2016). Therefore, including physiologically relevant growth media in the diagnostic workup may help to bridge the gap between in vitro and in vivo, improving the detection of fastidious microorganisms. Recently, a synthetic synovial fluid (SSF2) medium that supports the growth of a range of clinically relevant PJI isolates was developed. In SSF2, microorganisms adopt a biofilm-like aggregate phenotype similar to the one observed in vivo in infected joints (De Bleeckere et al., 2025).

The time frame in which a diagnosis can be made is crucial as well, in order to provide timely treatment and minimize healthcare costs (Premkumar et al., 2021). In this regard, isothermal microcalorimetry (IMC) may represent a promising alternative to CC, allowing rapid, real-time, non-destructive detection of viable microorganisms in a sample by measuring heat produced as a byproduct of microbial metabolism (Braissant et al., 2010). IMC significantly reduces the time to detection (TTD) compared to CC in various clinical settings, including urinary tract infections (Bonkat et al., 2012, 2013) and fracture-related infections (Cichos et al., 2022). Recent findings show that IMC outperformed culture-based diagnostics in detecting pathogens in SF samples from patients with PJI, with a shorter TTD and higher diagnostic sensitivity and accuracy (Cichos et al., 2023).

The goal of the present study was to explore whether the integration of SSF2 as an alternative culture medium and IMC can enhance diagnostic accuracy and reduce TTD in PJI. To this end, we analyzed 79 SF samples collected from 78 patients with suspected PJI. Each sample was investigated using multiple diagnostic approaches, including CC and culturing in SSF2, allowing for the comparison of detection rates. Additionally, all samples were investigated using IMC to evaluate its ability to shorten the TTD by assessing how fast it can identify samples containing viable microorganisms.

## 2 Materials

### 2.1 Collection of SF samples

A total of 79 SF samples were collected from 78 patients suffering from suspected PJI at Ghent University Hospital (Table S1 in the Supplement). This study was approved by the Ethics Committee of Ghent University Hospital (registration number BC-08886). SF was collected in sterile containers. To avoid contamination during sampling, the skin of the patient was routinely disinfected with polyvidone-iodine 10 % solution or with chlorhexidine digluconate 70 % alcohol. Samples were either processed immediately (in the case of CC) or stored at - 80 °C. 

### 2.2 Pathogen detection and identification using CC

Samples were cultured on blood agar (trypticase soy agar with 5 % sheep blood) and chocolate agar (GC II agar with IsoVitaleX) (aerobic, 35 °C, 7 d), in fluid thioglycolate medium (FTM) (aerobic, 35 °C, 10 d), and on Schaedler agar (Schaedler Anaerobe Agar, 5 % sheep blood, haemin, vitamin K1) (anaerobic, 35 °C, 10 d). The presence of growth was evaluated twice daily. In addition, samples were inoculated into blood-culture bottles (BCBs). Anaerobic bottles were inoculated first and incubated at 35 °C for 5 d; if additional fluid was available, aerobic bottles were inoculated and incubated at 35 °C for 5 d. FTM cultures and BCB that showed growth were subcultured on blood agar (aerobic) and Schaedler agar (anaerobic) and were incubated overnight at 35 °C under aerobic conditions and/or anaerobic conditions. Microbial colonies were identified using matrix-assisted laser desorption/ionization mass spectrometry (MALDI-TOF MS), with high-confidence identifications assigned at log scores ≥ 2.00 (category A). For lower scores, additional steps were taken according to the decision tree presented in Fig. S1 in the Supplement. Depending on the species, score differences between top matches, and spectrum quality, manual spectrum review or re-spotting was undertaken. Every organism that grew in CC was included in the dataset, even if below the clinical reporting threshold. For samples with a limited volume, not all media could be inoculated, resulting in incomplete data for these samples. As experiments with SSF2 and IMC were carried out on banked samples, clinicians were not aware of the results obtained with these approaches at the time of treatment, which was guided by CC outcomes.

### 2.3 Pathogen detection and identification using SSF2 medium

SSF2 medium was prepared as previously described (De Bleeckere et al., 2025). For each SF sample, 100 µL was inoculated in 900 µL SSF2 in a 24-well plate, with 1 well of SSF2 serving as control. Plates were incubated for 10 d at 37 °C under aerobic and anaerobic conditions (5 % H_2_, 5 % CO_2_, 90 % N ${}_{2})$ . Growth was assessed daily by visual inspection without opening the plates. Only upon observation of visible growth were plates opened and was 100 µL taken out and plated on Columbia blood agar (CBA; 5 % sheep blood) and incubated for 48 h at 37 °C under either aerobic or anaerobic conditions, corresponding to the environment in which initial growth was detected. If no growth was observed on CBA, 100 µL was plated on chocolate agar (if growth in SSF2 occurred in aerobic conditions) or on Schaedler agar (Schaedler Anaerobe Agar, 5 % sheep blood, haemin, vitamin K1) (if growth in SSF2 occurred in anaerobic conditions). Plates were then incubated either aerobically or anaerobically at 37 °C for 48 h. Resulting cultures were subcultured on CBA (chocolate or Schaedler agar when no growth was observed on CBA), and colonies were identified by MALDI-TOF MS.

### 2.4 Whole-genome sequencing of *M. luteus* and *K. rhizophila* isolates

DNA extraction was performed using the EZ2 PowerFecal Pro DNA/RNA Kit on the EZ2 Connect instrument (Qiagen, Hilden, Germany). Genomic libraries were prepared using the Illumina DNA PCR-free library preparation kit (Illumina, San Diego, CA, USA). Sequencing was carried out on a NovaSeq X Plus instrument (Illumina) using the NovaSeqX 1.5B sequencing kit with paired end reads ( 2×50 bp). Raw reads were quality filtered and trimmed using Trimmomatic v0.39 (Bolger et al., 2014). De novo assemblies were generated with SPAdes v3.15.4 using isolate-specific settings (Prjibelski et al., 2020). Sequencing data can be found in the NCBI BioProject database (https://www.ncbi.nlm.nih.gov/bioproject/, last access: 2 March 2026) under accession numbers PRJNA1399698 (*M. luteus*) and PRJNA1399699 (*K. rhizophila*). Core genome MLST (cgMLST) analysis was performed with chewBBACA v2.5.5 using in-house-developed cgMLST schemes (*M. luteus*: 1501 loci, *K. rhizophila*: 1555 loci) (Silva et al., 2018). Minimum spanning trees (MSTs) were created with PHYLOViZ Online (Ribeiro-Gonçalves et al., 2016). Correlation plots were generated in R v4.4.1, including only loci shared by all isolates.

### 2.5 Validation of IMC as a detection method

Prior to analyzing SF samples with IMC using the calScreener device, we validated the system's sensitivity using six PJI isolates (i.e., *Staphylococcus aureus *SAU060112, *Escherichia coli *UZ300822-0412-1, *Candida albicans *UZ221012-3305-1, *Staphylococcus epidermidis *HD05-1ST2, *Pseudomonas aeruginosa *UZ230406-3644-1, and *Cutibacterium acnes *CCUG48138). Pure and overnight cultures were incubated at 37 °C under appropriate aerobic or anaerobic conditions (Table S2). For each strain, we determined the correlation between optical density (OD) and the number of CFU mL^−1^; this allowed us to prepare inocula with the desired concentrations. To achieve final inocula of 10, 100, and 1000 CFU mL^−1^, overnight cultures were diluted in the appropriate growth media (BHI for most isolates; FTM was used for *C. acnes*) and transferred, using reversed pipetting, to plastic inserts compatible with the titanium cups of the calScreener. To confirm accuracy of the dilutions, all suspensions were plated, and the number of CFUs was calculated after incubation. After sealing the cups, they were placed in a 48-well calPlate and inserted into the instrument. The heat flow was measured at 37 °C for 96 h, and data were analyzed using calView software, with signals ≥ 5 µW defined as positive. The time at which this occurred was defined as the time to activity (TTA), serving as a measure for the TTD. Experiments were performed in biological duplicate (each consisting of two technical replicates).

### 2.6 Detection of microorganisms in SF using IMC

To minimize host-derived interference and enhance microbial recovery, all samples were first treated with 0.25 % trypsin EDTA. Briefly, 100 µL of each sample was centrifuged in a 96-well plate (3700 rpm, 15 min), whereafter the supernatant was removed, and 100 µL of 0.25 % trypsin EDTA was added to the pellet. Plates were incubated for 20 min at 37 °C and centrifuged again at 3700 rpm for another 15 min. After removing the trypsin, pellets were resuspended in 100 µL 0.9 % ( w/v ) NaCl. Subsequently, 30 µL was added to 270 µL of BHI, and 40 µL was added to 360 µL FTM by reverse pipetting (in plastic inserts placed in the titanium cups). After sealing the cups, the 48-well calPlate was inserted into the instrument, and heat flow was measured at 37 °C for 96 h and monitored using calView software. To explore the combined use of SSF2 and IMC, a subset of SF samples ( n=12 ; Fig. S2A) was analyzed in SSF2 (total volume of 300 µL ) in the calScreener, according to the protocol described above. All experiments were performed in technical quadruplicate. A sample was considered positive in a given medium if minimally two out of four replicates exceeded the threshold of 5 µW .

### 2.7 Statistical analysis

To compare TTA values obtained in BHI and FTM, for the SF samples that were positive in both media, a paired-sample t test was performed for normally distributed data, and a non-parametric Wilcoxon signed rank test was used for non-normally distributed data. Normal distribution was assessed using the Shapiro–Wilk test. For the subset of SF samples tested in all three media (BHI, FTM, and SSF2), a non-parametric Friedman test was conducted on the TTA values to assess overall differences between the media, followed by a Wilcoxon signed rank test for post hoc pairwise comparisons. The relationship between IMC and BCB detection times was evaluated using linear regression (Pearson correlation), after confirming assumptions of normality and linearity. Statistical analyses were performed using SPSS Statistics software (version 28) and GraphPad Prism (version 10.6.0 (796)).

## 3 Results

### 3.1 The overall positivity rate was the highest after culturing in SSF2

**Figure 1:**
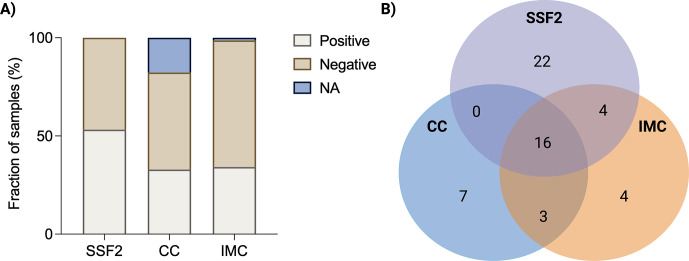
Summary of results obtained with different diagnostic approaches. **(A)** Overview of the fraction of SF samples that yielded a positive or negative result with the different diagnostic approaches (or for which data were not available; NA). After culturing in SSF2, 53.2 % (42/79) of samples were positive; after CC, 32.9 % (26/79) of samples were positive; and using IMC, 34.2 % (27/79) of samples were positive. **(B)** Venn diagram showing the number of positive samples as determined by CC (blue), SSF2 (purple), and IMC (orange) for the 56 SF samples that yielded a positive result with at least one approach.

After subjecting 79 SF samples to CC and culturing in SSF2, microorganisms in positive samples were identified, and detection rates were calculated. In addition, to evaluate the added value of IMC, each sample was inoculated in BHI and FTM and analyzed in the calScreener, after which positivity rates were compared. An overview of all samples that yielded a positive result with at least one technique ( n=56 ) is provided in Table 1. The highest positivity rate was observed after culturing in the SSF2 medium (53.2 %), followed by IMC (34.2 %) and CC (32.9 %) (Fig. 1A). Identification after CC revealed that 11.1 % of the positive samples were polymicrobial; these numbers were higher after culturing in SSF2 (50.0 %) (Table 1). Only 28.6 % of the positive samples were positive using all three approaches (Fig. 1B), and a substantial fraction of the positive cases (39.3 %) was exclusively detected after culturing in SSF2. There were also samples that only yielded positive results with other approaches, i.e., 12.5 % with CC only and 7.1 % with IMC only. The remaining positive samples yielded a positive result with two approaches (12.5 %).

**Table 1:** Results obtained with different diagnostic approaches for the samples for which at least one of the three diagnostic approaches yielded a positive result, along with organism(s) identified. NA: not available due to limited amount of SF available for analysis.

Sample	SSF2	Organism(s)	CC	Organism(s)	IMC BHI	IMC FTM
Septort122	-		+	*S. epidermidis*	-	-
Septort124	+	*E. faecalis*	+	*E. faecalis*	+	+
Septort126b	-		NA		-	+
Septort130	+	*K. rhizophila, S. epidermidis, S. mitis oralis,* *S. hominis*	-		-	-
Septort131	+	*M. luteus, A. ursingii, S. hominis*	-		-	-
Septort132	+	*E. faecalis*	NA		+	+
Septort134	-		+	*Paenibacillus *sp.	-	-
Septort135	+	*S. lugdunensis*	+	*S. lugdunensis*	+	+
Septort137	+	*M. luteus, K. rhizophila*	-		-	-
Septort138	-		-		-	+
Septort139	+	*G. haemolysans*	NA		-	-
Septort140	+	*S. epidermidis, M. luteus, K. rhizophila*	NA		-	-
Septort145	+	*S. capitis*	-		-	-
Septort150	+	*S. epidermidis*	-		-	-
Septort152	+	*S. aureus*	+	*S. aureus*	+	+
Septort153	+	*S. epidermidis*	-		-	-
Septort155	+	*S. epidermidis*	+	*S. epidermidis*	+	+
Septort159	+	*S. lugdunensis*	+	*S. lugdunensis*	+	+
Septort162	+	*S. hominis, P. yeei*	-		-	-
Septort163	-		+	*C. parapsilosis*	-	-
Septort164	+	*M. luteus, S. salivarius*	-		-	-
Septort165	+	*P. agglomerans*	-		-	-
Septort167	+	*S. epidermidis*	+	*S. epidermidis*	+	+
Septort169	+	*S. capitis, K. rhizophila*	+	*S. capitis*	-	+
Septort171	-		+	*S. epidermidis*	-	+
Septort172	+	*M. luteus*	-		-	-
Septort176	+	*C. acnes*	-		-	-
Septort177	+	*S. hominis, M. luteus*	NA		-	-
Septort178	+	*S. epidermidis, S. mitis oralis, S. capitis,* *S. hominis*	NA		-	-
Septort180	+	*S. capitis*	-		-	-
Septort183	+	*M. luteus*	-		-	-
Septort187	+	*S. hominis, M. luteus*	-		-	-
Septort191	+	*E. coli, S. epidermidis, S. hominis*	+	*E. coli,* *S. epidermidis,*	-	+
Septort192	-		NA		-	+
Septort194	-		+	*C. acnes*	-	-
Septort195	+	*S. epidermidis, A. viridans*	NA		+	+
Septort196	+	*S. aureus, K. rhizophila, M. luteus*	NA		+	-
Septort197	+	*S. epidermidis*	+	*S. epidermidis*	-	+
Septort200	-		+	*S. hominis*	-	NA
Septort202a	+	*S. epidermidis, S. pasteuri*	-		-	-
Septort206	+	*S. epidermidis, S. xylosus*	NA		-	+
Septort211	-		+	*S. capitis*	-	-
Septort213	-		-		-	+
Septort218	+	*C. acnes*	-		-	-
Septort226	-		+	*K. oxytoca,* *S. epidermidis*	+	+
Septort229	+	*S. lugdunensis*	+	*S. lugdunensis*	+	+
Septort230	+	*S. epidermidis, S. warneri*	+	*S. epidermidis*	+	+
Septort231	-		+	*C. acnes*	-	-
Septort239	+	*S. caprae, M. luteus, S. epidermidis,* *R. mannitolytica*	+	*S. caprae*	+	-
Septort240	+	*E. faecalis*	NA		-	+
Septort242	+	*C. difficile, S. epidermidis, S. hominis,* *S. pasteuri*	+	*C. difficile*	-	+
Septort243	+	*M. luteus, S. epidermidis*	+	*S. epidermidis*	+	-
Septort245	+	*S. epidermidis*	+	*S. epidermidis, S. hominis*	-	+
Septort248	+	*S. epidermidis, K. rhizophila, M. luteus*	-		-	-
Septort250	-		+	*S. caprae*	+	+
Septort260	+	*S. vestibularis, S. salivarius*	-		-	-

### 3.2 Culturing in SSF2 recovered the highest diversity of organisms

**Figure 2:**
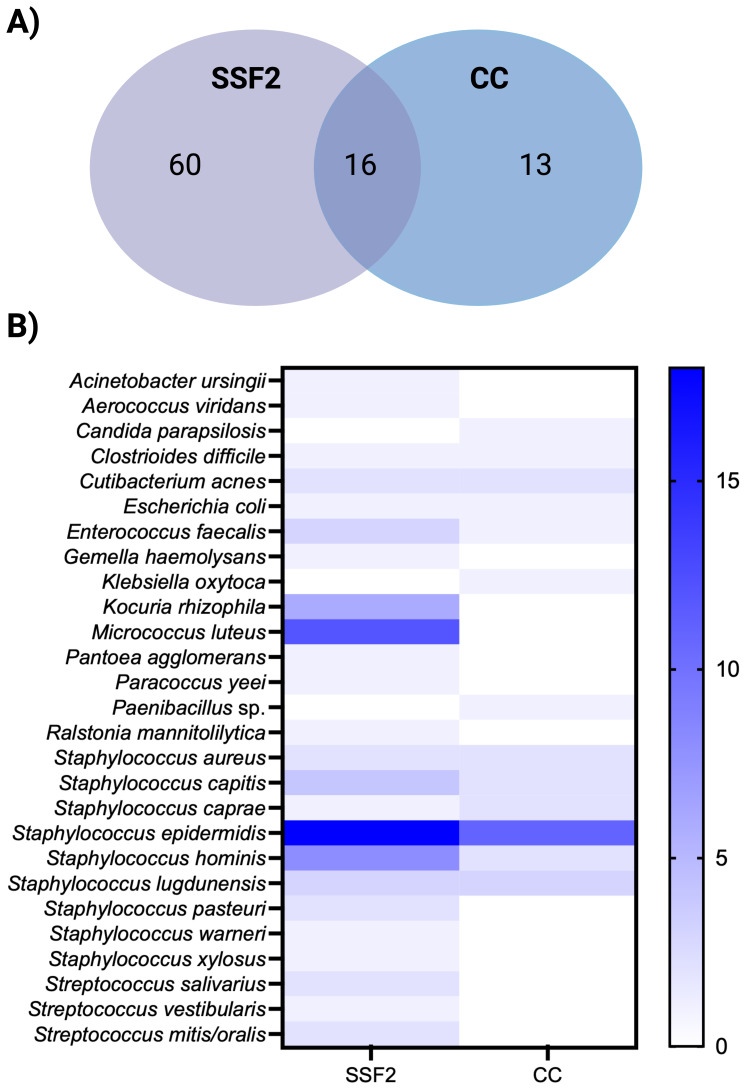
Organism-level detection within the positive samples after CC and/or culturing in SSF2 ( n=52 ). **(A)** Venn diagram showing the organism-level concordance between SSF2 culturing (purple) and CC (blue). **(B)** Organism-by-approach heatmap of detection rates. Rows list the organisms identified in the SF samples; columns represent the two diagnostic approaches. Cell shading ranges from white (not detected) to dark blue (highest number of detection).

Only organisms detected after CC and culturing in SSF2 were included in the organism-level comparison, as organisms in IMC-positive samples were not identified. A total of 52 samples tested positive after CC and/or culturing in SSF2. In total, 89 organisms were identified in these samples; either with a single approach ( n=73 ) or with both approaches ( n=16 ). The concordance in detecting the same organism in a sample between CC and culturing in SSF2 is shown in Fig. 2A. Only 18.0 % of the organisms were picked up by both approaches. Surprisingly, most organisms (82.0 %) were detected by one approach only, with the largest fraction (67.4 %) being only picked up after culturing in SSF2. An overview of how often an organism was detected by the different diagnostic approaches is shown in Fig. 2B. Some organisms were detected to the same extent with the two approaches (e.g., *C. acnes* and* Staphylococcus aureus*). In contrast, some organisms were detected exclusively using only one approach. For example, *Candida parapsilosis *and *Klebsiella oxytoca *were only recovered after CC, while *Kocuria rhizophila *and *Micrococcus luteus*, together with various coagulase-negative staphylococci and streptococci, were only detected after culturing in SSF2. When evaluating the detection frequency in SSF2, certain organisms were identified more frequently compared to CC.* Staphylococcus epidermidis *and *Staphylococcus hominis* were identified in 18 and 8 samples using SSF2 compared to 8 and 0 using CC, respectively. Notably, *K. rhizophila* and* M. luteus *were not just uniquely detected in SSF2, but also frequently, in 6 and 12 SF samples, respectively.

### 3.3 cgMLST indicates that *M. luteus* and *K. rhizophila* isolates are not clonally related

Given the remarkable prevalence of *M. luteus *and *K. rhizophila *detected in SSF2, the whole genome sequence of a selection of *M. luteus* ( n=9 ) and *K. rhizophila* ( n=5 ) isolates was determined, on which cgMLST analysis was performed. Pairwise allelic distances among isolates ranged from 250 to 420 alleles for *M. luteus *and 1200 to 1500 alleles for *K. rhizophila*. MSTs show long branch lengths between all isolates and no evidence of clustering (Fig. S2).

### 3.4 IMC allows rapid detection of PJI isolates in very low concentrations

**Figure 3:**
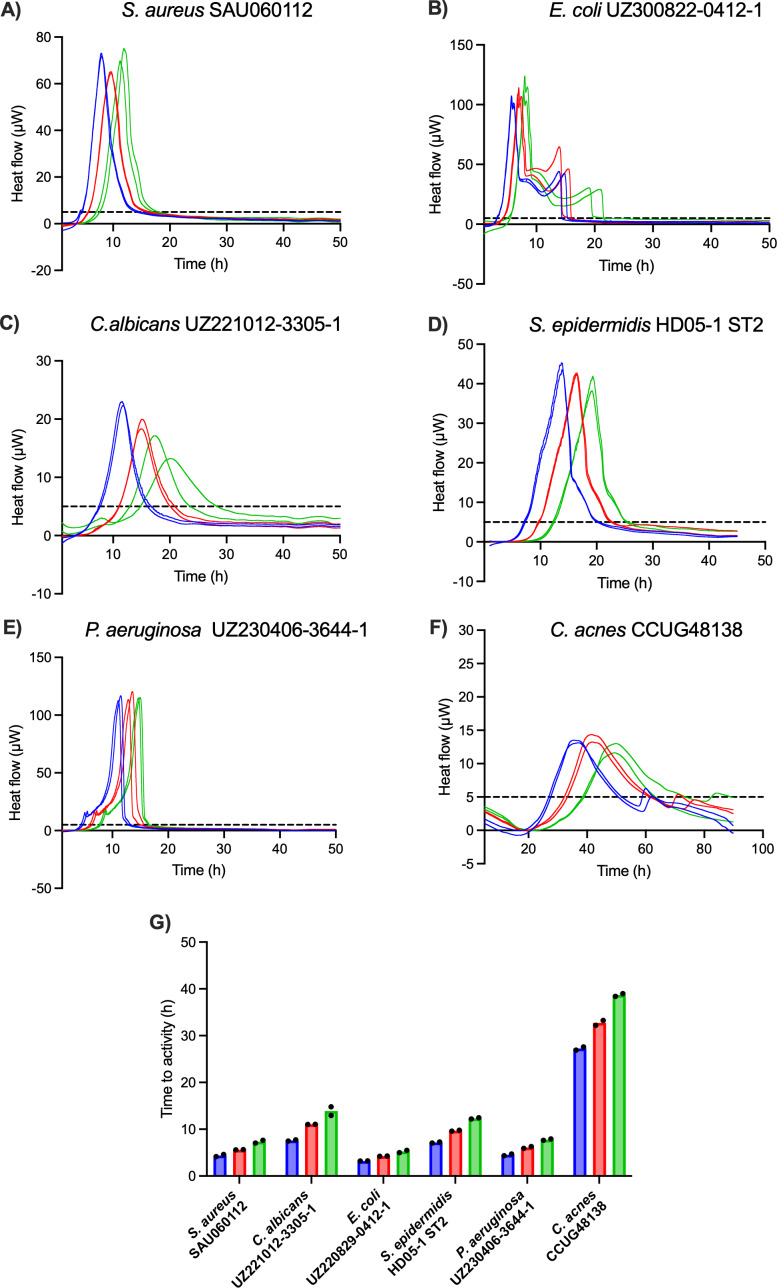
**(A–F)** Thermograms of six PJI isolates obtained with different inoculum sizes, i.e., 1000 CFU mL^−1^ (blue), 100 CFU mL^−1^ (red), and 10 CFU mL^−1^ (green). The threshold for activity (5 µW ) is indicated by the dashed black line. **(G)** Average TTAs for each isolate and inoculum size. All isolates were inoculated in BHI, except for *C. acnes*, which was inoculated in FTM. Each experiment was performed in biological duplicate ( n=2 ), and individual data points are presented by black dots.

To validate the sensitivity of IMC as a method of detection, six PJI isolates were inoculated in low concentrations (1000, 100, 10 CFU mL^−1^) in BHI (except for *C. acnes*, which was inoculated in FTM) in the calScreener, and heat production was measured for 96 h at 37 °C (Fig. 3). For each species, thermograms obtained with different inoculum sizes showed the same overall shape with a shift of the TTA and the peak (maximum metabolic activity) to later time points for lower inoculum sizes. All isolates were detected across all tested inoculum sizes in less than 15 h, except for *C. acnes*, for which the TTA was approximately 27, 33, and 39 h for 1000, 100, and 10 CFU mL^−1^ respectively. Fast-growing organisms were detected in under 8 h, even for the lowest inoculum. For instance, *Escherichia coli *showed the lowest TTA of less than 6 h for the inoculum of 10 CFU mL^−1^, while TTAs for the lowest inocula of *C. albicans *and *S. epidermidis* were below 14 and 13 h, respectively.

### 3.5 IMC allows us to rapidly identify samples that contain microorganisms

**Figure 4:**
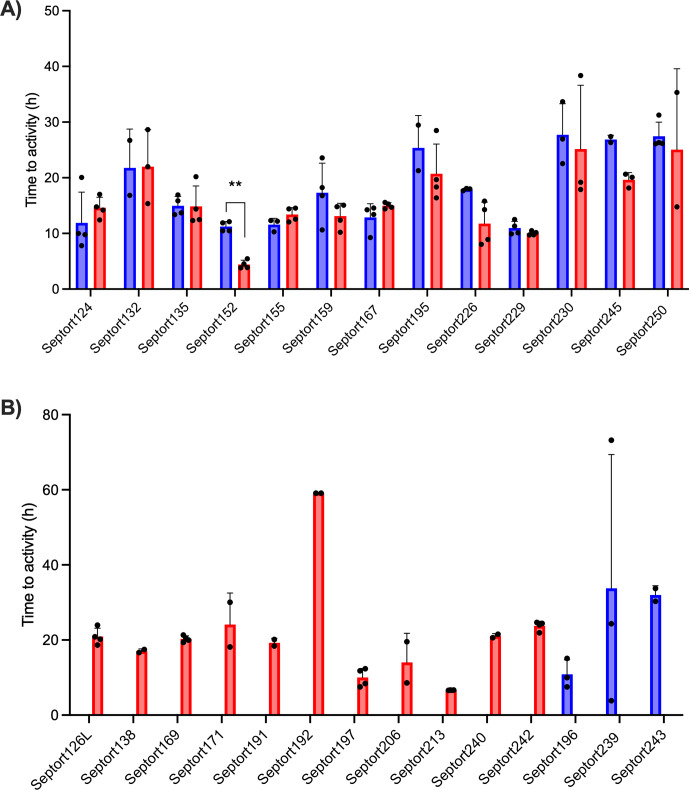
Average TTAs for all SF samples that resulted in a positive signal after IMC analysis. **(A)** TTA values for samples yielding a positive signal in both BHI (blue) and FTM (red) ( n=13 ) ${}^{**}\;p<0.01$ . **(B)** TTA values for samples yielding a positive signal in either BHI (blue) ( n=3 ) or FTM (red) ( n=11 ). Each experiment was performed in biological duplicate, with each containing two technical replicates ( n=4 ). A sample was considered positive in a given medium if ≥ 2 out of 4 replicates exceeded the threshold of 5 µW . Bars represent mean values of biological and technical duplicates ( n=2 –4); error bars indicate standard deviation, and individual data points are presented by black dots.

To decide which media to use for IMC analysis of the SF samples, a pilot study was performed on 12 samples, which were inoculated in BHI, FTM, and SSF2 and incubated in the calScreener (Fig. S3A). Only two samples (16.7 %) were positive in SSF2. The positivity rate in BHI and FTM was 25.0 % and 50.0 %, respectively. There was a statistically significant difference ( p=0.0273 ) in TTAs among the three media for both samples that yielded positive results in all media tested. Although a post hoc pairwise comparison was not possible due to the limited number of replicates in SSF2 ( n=2 ), higher TTAs were observed in SSF2 compared to BHI and FTM (Fig. S3B). For this reason, we omitted SSF2 as one of the media to be used for IMC and proceeded with BHI and FTM only. Overall, TTAs ranged from 3.85 to 73.19 h in BHI and from 3.81 to 59.08 h in FTM (Fig. 4). The overall positivity rate was the highest in FTM (88.9 %) compared to 59.3 % in BHI. Only 48.1 % of positive samples were positive in both media, while 11.1 % and 40.8 % were positive in BHI only or FTM only, respectively. The median TTA in BHI was 15.9 h (95 % CI, 12.5–18.2 h) versus 15.6 h in FTM (95 % CI, 14.4–18.3 h). For sample Septort152, the TTA was significantly lower in FTM than in BHI ( p=0.002 ). Other differences in TTA between media were not statistically significant ( n=8 ), or analysis was not possible due to the limited number of replicates ( n=2 ).

### 3.6 Comparison of the time to detect microbial activity with IMC and BCB

We subsequently compared the time to detect microbial activity in samples that were both IMC and BCB positive ( n=16 ) (Fig. S4A). Only these two approaches were compared in terms of detection times, as they allow for continuous monitoring, unlike the other approaches. For these 16 samples, detection times ranged from 11.0 to 33.8 h in BHI and 4.4 to 25.2 h in FTM for IMC and from 7.6 to 26.9 h for BCB. Using IMC, median detection times in BHI and FTM were 16.2 h (95 % CI, 11.8–27.2 h) and 14.9 h (95 % CI, 13.1–23.8 h), respectively; the median detection time in BCB was 18.1 h (95 % CI, 15.2–20.20 h). For 62.5 % of these samples, the detection time was the shortest using IMC. We observed a statistically significant correlation between the detection time with IMC in FTM on the one hand and growth in BCB on the other ( $R^{2}=0.5046$ ; p=0.0030 ). The correlation between the detection times with IMC in BHI and growth in BCB was not significant ( $R^{2}=0.3121$ ; p=0.0590 ) (Fig. S4B).

### 3.7 Distinct thermogram profiles reflect intra- and inter-sample variability

**Figure 5:**
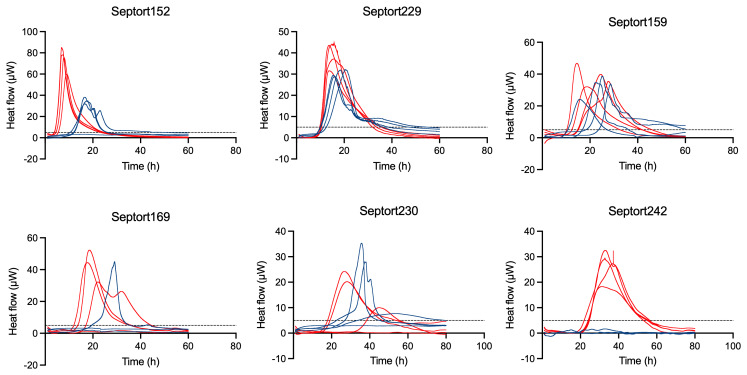
Thermograms of six SF samples. The threshold for activity (5 µW ) is indicated by the dashed black line. Samples were inoculated in BHI (blue) and FTM (red) in technical quadriplate ( n=4 ).

For each of the 27 IMC-positive samples, thermograms obtained in FTM differed from those obtained in BHI, likely related to medium-dependent metabolism. A selection of six IMC-positive samples was made to illustrate corresponding thermograms (Fig. 5). For 25.9 % of these samples, replicate thermograms were highly similar (e.g., Septort152, Septort229, and Septort242). In contrast, for 33.3 % of these samples, replicate thermograms obtained in the same medium varied in TTA and/or shape (e.g., Septort159, Septort169, and Septort230). Some samples were only positive in FTM (40.7 %) (e.g., Septort242) or in BHI (11.1 %). Lastly, in 66.6 % of the positive samples, not all replicates produced a detectable signal (e.g., Septort169 and Septort230).

## 4 Discussion

In this study we evaluated (i) whether the use of SSF2 as a culture medium improved microbial recovery from SF and (ii) whether IMC reduced the time to detect microbial activity. Culturing in SSF2 yielded the highest positivity rate, followed by IMC and CC. Only 28.6 % of the positive samples were positive with all three approaches, and, remarkably, 39.3 % of all positive samples were only identified as such after culturing in SSF2, indicating that a substantial number were not picked up by CC and/or IMC. However, CC and IMC also identified a smaller subset of positive samples not identified using SSF2, highlighting the complementary value of combining diagnostic approaches. Interestingly, CC and culturing in SSF2 also varied in their ability to identify samples that are polymicrobial: while CC identified 11.1 % of samples as polymicrobial, SSF2 revealed that 50.0 % of the samples contained multiple organisms. This is remarkable, given that previous studies have reported polymicrobial prevalence in PJI at 15 %–30 % (Flurin et al., 2019; Fröschen et al., 2022; Li et al., 2021). These findings suggest that mimicking the synovial microenvironment during culture allows for the identification of more polymicrobial samples. The low-nutrient nature of SSF2 may help prevent fastidious organisms from being overgrown by fast-growing ones. Moreover, the transition from the in vivo microenvironment to a physiologically relevant in vitro medium may limit the induction of the viable but non-culturable (VBNC) state (Pazos-Rojas et al., 2024), thereby improving recovery. Analysis of the microbiological composition of the SF samples revealed that *S. epidermidis *was the most commonly detected organism across both approaches, followed by other coagulase-negative staphylococci, in line with other studies (Arciola et al., 2005; Barbero Allende et al., 2024; Flurin et al., 2019; Fröschen et al., 2022; Haraldsdóttir et al., 2025; Shabana et al., 2023). Certain organisms were more frequently recovered in SSF2, including *S. epidermidis, S. hominis, M. luteus*, and *K. rhizophila*, with the latter two only detected in SSF2. Although *Kocuria *spp. and *Micrococcus *spp. are often dismissed as laboratory or skin contaminants (Kandi et al., 2016), case reports suggest they may have clinical relevance in PJI (Baumbach et al., 2018; Koenemann et al., 2023; McAleese et al., 2023). To address this, cgMLST analysis was performed on a selection of sequenced *M. luteus *and *K. rhizophila* isolates detected in SSF2, revealing large allelic distances between isolates and absence of clustering, ruling out genetical relatedness and making a common source highly unlikely. Additional studies will be needed to investigate whether these species have any clinical relevance in PJI. The distinction between skin contaminants and true pathogens remains challenging in the context of PJI, particularly for low-virulence organisms, as infection may arise from accidental intraoperative inoculation with skin commensals (Boyle et al., 2018). However, systematically dismissing such organisms as contaminants may lead to underestimating their clinical relevance.

Our data show that PJI isolates can rapidly be detected using IMC, even when present in very low numbers, with the TTA increasing as the bacterial load decreases. Importantly, 10 CFU mL^−1^ of *C. acnes* was detected by IMC in 39 h, while the literature reports the TTD using CC ranges from 2–14 d, depending on inoculum size and medium composition (Butler-Wu et al., 2011; Jeverica et al., 2020). When evaluating IMC performance in analyzing SF samples, FTM generally outperformed BHI, likely due to its support of both aerobic and anaerobic growth. However, some samples were positive in BHI only, supporting the combined use of both media in IMC. Within the 96 h measurement window, both BHI and FTM outperformed SSF2 when it came to detecting positive samples. This does not imply that IMC is unable to detect growth in SSF2 but likely reflects reduced metabolic activity due to the nutritional composition of SSF2. These initial experiments indicate that the combined use of SSF2 and IMC does not offer an advantage in reducing TTD, and for this reason, all further IMC experiments were conducted in BHI and FTM only. Median detection times with IMC were higher (15.9 h in BHI and 15.6 h in FTM) than the 2–12 h range previously reported for PJI samples (Cichos et al., 2022, 2023; Morgenstern et al., 2020; Christensen et al., 2024). Notably, detection times obtained with BCB were within a similar range to those obtained with IMC, suggesting that both approaches may offer similar benefits in terms of rapid detection (Sanabria et al., 2019). IMC was not compared to other culture-based approaches in terms of detection times, as these were not continuously monitored, possibly leading to bias. Nevertheless, previous work reports that CC typically requires ≥ 50 h (2–3 d) for detection (Cichos et al., 2022, 2023; Morgenstern et al., 2020), supporting our finding that both IMC and BCB outperform such conventional cultures. Replicate thermograms often shared the same overall shape, although TTAs sometimes differed. We suspect that this is indicative of an uneven bacterial distribution in the sample, likely due to the presence of aggregates that form in SF (Pestrak et al., 2020; Staats et al., 2021, 2022). In some cases, replicate thermograms showed different shapes, likely pointing to different organisms being detected in different replicates. Combined with the uneven distribution of organisms discussed above, this suggests that these samples are polymicrobial. Although direct confirmation of the polymicrobial nature of these cultures is not possible (as microorganisms detected in IMC-positive samples were not identified), the corresponding SSF2 cultures did recover multiple microorganisms from these samples. In two-thirds of the IMC-positive samples, not all replicates yielded a signal, which could be due to uneven distribution of cellular aggregates, (very) low bacterial loads, and/or stochastic variation. Prior studies have indeed shown that heterogeneously distributed aggregates in clinical samples complicate detection, certainly when present in low microbial loads (Jakobsen et al., 2025). Although there are indications that heat flow profiles generated by IMC can be used to distinguish microorganisms (Di Luca et al., 2019; Morgenstern et al., 2020), it has not yet been validated for accurate species-level identification. Consequently, organisms detected in IMC-positive samples still need to be identified using other approaches. However, since IMC is a non-destructive technique, downstream analyses such as NGS or MALDI-TOF MS can be performed on the same sample or its subculture.

One of the limitations of this study is the difference in sample handling between methods. While samples analyzed in the clinical microbiology lab were processed immediately upon collection, those subjected to culturing in SSF2 and IMC were frozen immediately after collection and thawed once prior to culturing in SSF2 and once prior to inoculation in the calScreener (i.e., a maximum of two freeze–thaw cycles) (Cabello-Olmo et al., 2020; Cuthbertson et al., 2015). This could contribute to the longer detection times using IMC observed in this study compared to previous work (Cichos et al., 2022, 2023; Morgenstern et al., 2020; Christensen et al., 2024). Another limitation is the different sample volume of SF that was used across different approaches. Using a lower volume potentially impacts detection rates, particularly in samples with a low microbial load (Jakobsen et al., 2025). Additionally, due to sample volume restrictions, 16.7 % of the samples were unavailable for CC and 1.3 % for IMC. Furthermore, all approaches remain vulnerable to false positives due to skin or laboratory contaminants or resident joint microbiota (Pattyn et al., 2017; Vrancianu et al., 2023), underscoring the need for aseptic sampling and appropriate controls, along with a better understanding of the native joint microbiome (Fernández-Rodríguez et al., 2023). Finally, the study would have benefited from a more appropriate selection of the study population, for example, by stratifying participants according to established diagnostic criteria such as the EBJIS definition for PJI, which would have made it easier to assess the clinical relevance of the microorganisms detected.

As a proof-of-concept study, the present work was designed to explore the potential of SSF2 and IMC as alternative microbiological diagnostic approaches, rather than to establish or validate clinical diagnosis in PJI. Consequently, microbiological findings were not linked to other data or patient outcomes. In line with current PJI diagnostic guidelines, which require concordant positivity across multiple sample types for microbiological confirmation, future research should include a broader range of sample types per patient, and microbiological results should be interpreted alongside clinical features and relevant biomarkers to help distinguish true pathogens from contaminants (McNally et al., 2021). Future studies investigating the performance of the SSF2 medium and IMC should include comparison to NGS-based techniques and comparison to detection after sonication of SF and tissue samples (Jakobsen et al., 2025; Kato et al., 2023). Follow-up studies with larger, multicenter patient cohorts and more carefully defined study populations are needed to determine whether the use of SSF2 and IMC ultimately would lead to better patient outcomes.

## 5 Conclusions

In conclusion, culturing in SSF2 uncovered the greatest number and diversity of organisms. IMC using BHI and FTM allowed identification of positive samples in less than 16 h, reducing the time to detect microbial activity in a clinical sample by 1–2 d compared to routine culture. Culturing in SSF2 and IMC are complementary approaches, as IMC can rapidly detect the presence of fast-growing organisms in a sample, while SSF2 can pick up fastidious organisms in samples that otherwise would be labeled as culture negative. These findings highlight SSF2 culture and IMC as promising tools toward faster and more accurate PJI diagnostics.

## Supplement

10.5194/jbji-11-149-2026-supplement The supplement related to this article is available online at https://doi.org/10.5194/jbji-11-149-2026-supplement.

## Data Availability

Sequencing data are available in the NCBI BioProject database (https://www.ncbi.nlm.nih.gov/bioproject/, last access: 2 March 2026), with accession numbers PRJNA1399698 and PRJNA1399699. All other data are available from the authors upon request.
