# Supplementary material for: Advancing diagnostics in suspected periprosthetic joint infections using synthetic synovial fluid and microcalorimetry
Source: J Bone Jt Infect. 2026 Mar 11;11(2):149–60. doi: 10.5194/jbji-11-149-2026 (PMC13034168; doi:10.5194/jbji-11-149-2026)
Supplement: The supplement related to this article is available online at https://doi.org/10.5194/jbji-11-149-2026-supplement. [file jbji-11-149-2026-supplement.pdf]

Supplement of J. Bone Joint Infect., 11, 149–160, 2026
https://doi.org/10.5194/jbji-11-149-2026-supplement
© Author(s) 2026. CC BY 4.0 License.

JBJI
Journal of Bone and Joint Infection
Open Access

Supplement of

Advancing diagnostics in suspected periprosthetic joint infections using synthetic synovial fluid and microcalorimetry

Amber De Bleeckere et al.

Correspondence to: Tom Coenye (tom.coenye@ugent.be)

The copyright of individual parts of the supplement might differ from the article licence.

Supplementary

Table S1. All synovial fluid samples included in this study, along with patient and procedure characteristics.

Sample	Joint	Procedure	Prosthesis
Septort117	Knee	Arthroscopy	spacer
Septort118	Knee	Arthroscopy	pTKR
Septort122	Hip	Arthroscopy	rTHP
Septort124	Knee	Open DAIR	rTKR
Septort126a	Knee	Arthroscopy	pTKP
Septort126b	Knee	Arthroscopy	pTKP
Septort130	Knee	Arthroscopy	pTKR
Septort131	Knee	Arthroscopy	pTKR
Septort132	Knee	Open first stage	pTKR
Septort134	Knee	Arthroscopy	pTKR
Septort135	Knee	Arthroscopy	rTKR
Septort137	Knee	Arthroscopy	rTKP
Septort138	Knee	Arthroscopy	rTKR
Septort139	Knee	Arthroscopy	pTKR
Septort140	Knee	Arthroscopy	pTKR
Septort142	Knee	Arthroscopy	pTKR
Septort144	Hip	Open first stage	pTHP
Septort145	Knee	Open first stage	pTKR
Septort150	Knee	Arthroscopy	pTKP
Septort152	Hip	Arthroscopy	rTHP
Septort153	Knee	Arthroscopy	pTKR
Septort155	Knee	Arthroscopy	TKR
Septort157	Knee	Arthroscopy	TKP
Septort159	Knee	Open	rTKP
Septort162	Knee	Arthroscopy	rTKR
Septort163	Hip	Open first stage	pTHR
Septort164	Knee	Arthroscopy	pTKP
Septort165	Knee	Arthroscopy	pTKR
Septort167	Knee	Open DAIR	rTKP
Septort169	Knee	Open	pTKR
Septort170	Knee	Arthroscopy	pTKR
Septort171	Knee	Arthroscopy	pTKP
Septort172	Knee	Arthroscopy	pTKP
Septort174	Knee	Arthroscopy	pTKR
Septort176	Hip	Arthroscopy	pTHP
Septort177	Knee	Arthroscopy	rTKP
Septort178	Knee	Arthroscopy	rTKP
Septort180	Knee	Arthroscopy	pTKP
Septort183	Knee	Arthroscopy	pTKR
Septort184	Knee	Arthroscopy	rTKR

Septort188	Hip	Arthroscopy	Resurfacing prosthesis
Septort191	Hip	Open	rTHP
Septort192	Knee	Arthroscopy	pTKP
Septort194	Knee	Arthroscopy	pTKP
Septort195	Knee	Open	pTKP
Septort196	Hip	Open partial	rTHP
Septort197	Knee	Open first stage	rTKP
Septort200b	Shoulder	Arthroscopy	rTSP
Septort201	Knee	Arthroscopy	pTKP
Septort202a	Knee	Arthroscopy	rTKP
Septort206	Hip	Open	rTHP
Septort207	Knee	Arthroscopy	rTKP
Septort209	Knee	Open first stage	pTKP
Septort211	Knee	Arthroscopy	rTKP
Septort213	Knee	Open first stage	pTKP
Septort215	Knee	Arthroscopy	rTKR
Septort218	Knee	Arthroscopy	pTKP
Septort226	Knee	Open DAIR	rTKP
Septort228	Knee	Arthroscopy	pTKR
Septort229	Knee	Arthroscopy	pTKR
Septort230	Knee	Arthroscopy	rTKP
Septort231	Knee	Arthroscopy	rTKR
Septort234	Knee	Arthroscopy	pTKP
Septort236	Knee	Arthroscopy	pTKR
Septort237	Knee	Arthroscopy	pTKR
Septort239	Knee	Arthroscopy	rTKP
Septort240	Hip	Open first stage	rTHP
Septort241	Knee	Arthroscopy	pTKR
Septort242	Knee	Open mix FRI + PJI	Spacer Knee
Septort243	Hip	Arthroscopy	rTHP
Septort244	Hip	Arthroscopy	pTHP
Septort245	Hip	Open DAIR	rTHP
Septort248	Knee	Open first stage	pTKP
Septort249	Knee	Arthroscopy	pTKR
Septort250	Knee	Open first stage	rTKR
Septort255	Knee	Arthroscopy	rTKR
Septort259	Knee	Arthroscopy	pTKP
Septort260	Knee	Open	rTKR
Septort262	Knee	Arthroscopy	pTKP

DAIR, Debridement, Antibiotics, and Implant Retention; FRI, Fracture related Infection; pTHP, primary Total Hip Prosthesis; pTKP, primary Total Knee Prosthesis; pTHR, primary Total Hip Replacement; pTKR, primary Total Knee Replacement; PJI, Periprosthetic Joint Infection; rTHP, revision Total Hip Prosthesis; rTKP, revision Total Knee Prosthesis; rTKR, revision Total Knee Replacement

Table S2. Isolates used for the validation of IMC as a detection method

Species	Designation	Origin	Source (Reference)	Solid culture media	Liquid culture media
S. aureus	SAU060112	Prosthetic components, psoriatic arthritis	T.R. Thomsen (Xu et al. (1))	TSA	MHB
S. epidermidis	HD05-1 ST2	Human synovial fluid, knee	H. Rohde (Both et al. (2))	TSA	MHB
C. acnes	CCUG48138	Human synovial fluid	CCUG	RCM agar	RCM
E. coli	UZ220829-0412-1	Human synovial fluid	Own isolate	TSA	MHB
P. aeruginosa	UZ230406-3644-1	Chronic osteomyelitis	Own isolate	TSA	MHB
C. albicans	UZ221012-3305-1	Human synovial fluid	Own isolate	SAB agar	SAB broth

MHB, Mueller Hinton Broth (Lab M, Moss Hall, UK); RCM, Reinforced Clostridial Medium (Lab M); SAB, Sabouraud dextrose medium (Lab M); TSA, Tryptic Soy Agar (Neogen, Heywood, UK).

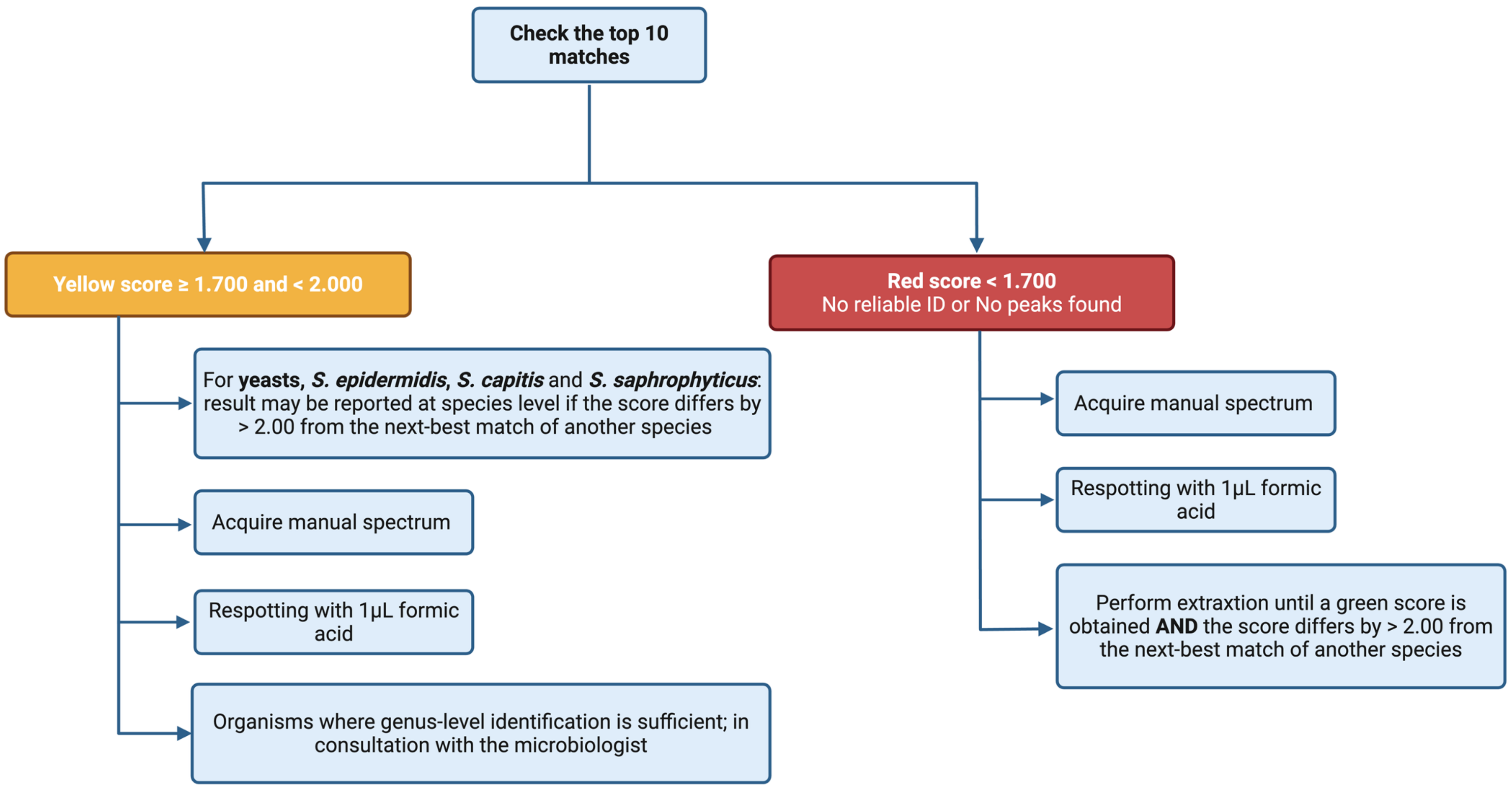

Figure S1. Decision tree for the interpretation of the MALDI-TOF log-scores.

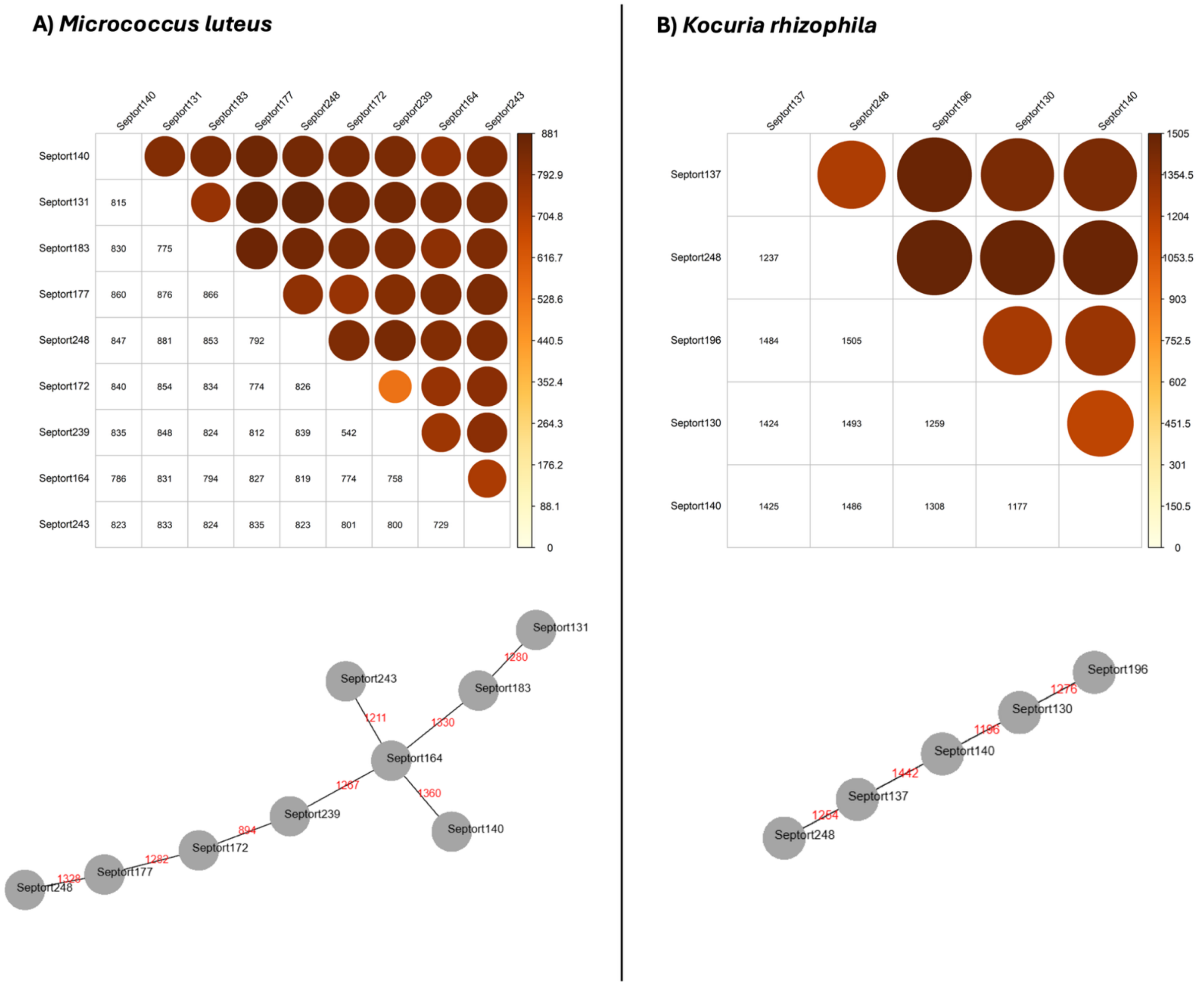

Figure S2. cgMLST analysis of *M. luteus* (A) and *K. rhizophila* (B) isolates detected in SSF2. Upper panels show pairwise allelic distance matrices, with circle size and color intensity representing the number of allelic differences between isolates. Corresponding MSTs are shown in lower panels, in which nodes represent individual isolates and edges are labeled with allelic distances between connected isolates.

A)

	SSF2	BHI	FTM
Septort130	-	-	-
Septort150	-	-	-
Septort152	+	+	+
Septort159	+	+	+
Septort162	-	-	-
Septort178	-	-	-
Septort191	-	-	+
Septort194	-	-	-
Septort202b	-	-	-
Septort213	-	-	+
Septort240	-	+	+
Septort242	-	-	+

B)

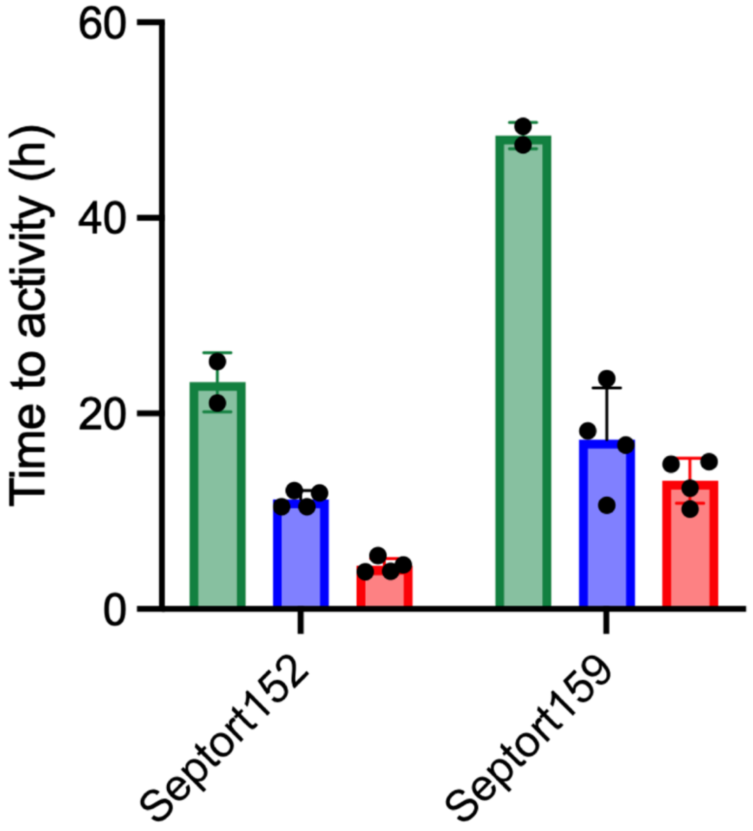

Figure S3. IMC results for subset of synovial fluid samples that were tested in all three media. (A) Overview of positivity outcomes in BHI, FTM and SSF2. (B) Average TTAs for all synovial fluid samples that resulted in a positive signal after IMC analysis in SSF2 (green), BHI (blue) and FTM (red). Each experiment was performed in biological duplicate, which was performed in technical duplicate (n=4). A sample was considered positive in a given medium if ≥ 2 out of 4 replicates exceeded the threshold of 5 μW. Bars represent mean values of biological and technical duplicates (n=2-4); error bars indicate standard deviation and individual datapoints are presented by black dots.

A)

Sample	CC	IMC	
	BCB	BHI	FTM
Septort124	10.5	11.9	14.6
Septort135	15.2	15.0	14.9
Septort152	7.6	11.2	4.40
Septort155	20.5	11.6	13.4
Septort159	14.2	17.3	13.1
Septort167	20.1	12.4	15.0
Septort169	17.7	negative	20.3
Septort171	21.5	negative	24.1
Septort197	20.2	negative	10.0
Septort226	15.5	17.9	11.8
Septort229	13.9	11.0	10.0
Septort230	26.9	27.7	25.2
Septort239	17.7	33.8	negative
Septort242	18.5	negative	23.8
Septort245	19.0	26.9	17.0
Septort250	21.0	27.5	25.0

B)

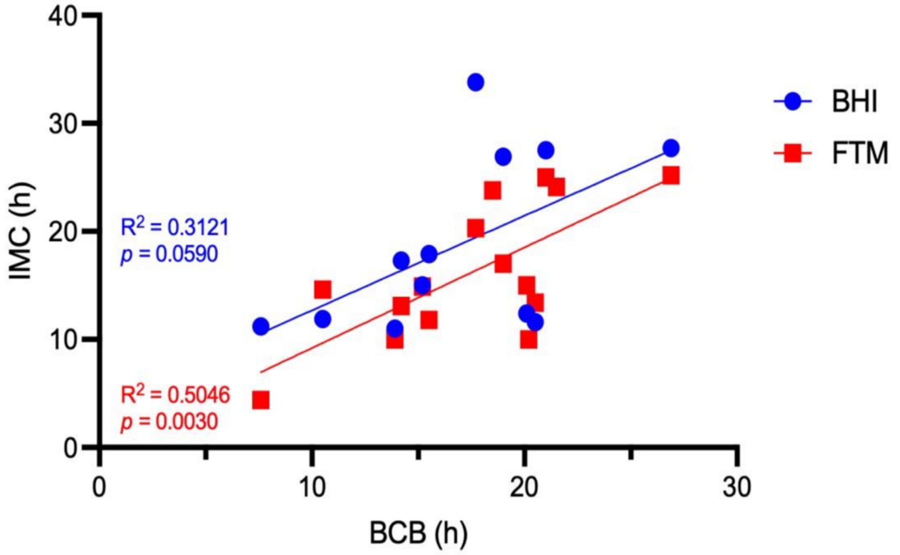

Figure S4. Comparison of the time to detect microbial activity in a sample using IMC versus BCB. (A) Table providing times to detect microbial activity (expressed in hours) with each approach, for all samples that were both CC- and IMC-positive (n=16). **(B)** Scatter plot shows relationship between the time to detect microbial activity (in hours) obtained with IMC, under two conditions (BHI; blue circles, FTM; red squares), and the time to detect microbial activity obtained with BCB for the samples that were both IMC- and BCB-positive (n=16). Linear regression lines are shown for both conditions, with corresponding correlation coefficient and *p*-value.

References

1. Xu Y, Maltesen RG, Larsen LH, Schønheyder HC, Le VQ, Nielsen JL, et al. In vivo gene expression in a Staphylococcus aureus prosthetic joint infection characterized by RNA sequencing and metabolomics: A pilot study. BMC Microbiol. 2016 May 5;16(1):1–12.
2. Both A, Huang J, Qi M, Lausmann C, Weißelberg S, Büttner H, et al. Distinct clonal lineages and within-host diversification shape invasive staphylococcus epidermidis populations. PLoS Pathog. 2021 Feb 5;17(2).
